# Methylenetetrahydrofolate reductase polymorphic variants C677T and A1298C in rectal cancer in Slavic population: significance for cancer risk and response to chemoradiotherapy

**DOI:** 10.3389/fgene.2023.1299599

**Published:** 2024-01-15

**Authors:** Aleksandra Stanojevic, Jelena Spasic, Mladen Marinkovic, Suzana Stojanovic-Rundic, Radmila Jankovic, Ana Djuric, Jerome Zoidakis, Remond J. A. Fijneman, Sergi Castellvi-Bel, Milena Cavic

**Affiliations:** ^1^ Department of Experimental Oncology, Institute for Oncology and Radiology of Serbia, Belgrade, Serbia; ^2^ Clinic for Medical Oncology, Institute for Oncology and Radiology of Serbia, Belgrade, Serbia; ^3^ Clinic for Radiation Oncology and Diagnostics, Department of Radiation Oncology, Institute for Oncology and Radiology of Serbia, Belgrade, Serbia; ^4^ Faculty of Medicine, University of Belgrade, Belgrade, Serbia; ^5^ Department of Biotechnology, Biomedical Research Foundation, Academy of Athens, Athens, Greece; ^6^ Department of Biology, National and Kapodistrian University of Athens, Athens, Greece; ^7^ Department of Pathology, The Netherlands Cancer Institute, Amsterdam, Netherlands; ^8^ Gastroenterology Department, Fundació Recerca Clínic Barcelona-Institutd’Investigacions Biomèdiques August Pi iSunyer, Centro de Investigación Biomédicaen Red de Enfermedades Hepáticas y Digestivas, Clínic Barcelona, University of Barcelona, Barcelona, Spain

**Keywords:** chemoradiotherapy, methylenetetrahydrofolate reductase, MTHFR C677T, MTHFR A1298C, rectal cancer, single nucleotide polymorphism

## Abstract

**Background:** Methylenetetrahydrofolate reductase (MTHFR) single nucleotide polymorphisms (SNPs) have been suggested as risk, prognostic, and predictive factors for colorectal cancer in various populations, but have not been validated so far. The aim of this study was to examine the association of *MTHFR* C677T (*rs1801133*) and A1298C (*rs1801131*) single nucleotide polymorphisms with the risk of rectal cancer as well as the response to neoadjuvant chemoradiotherapy (nCRT) based on 5-Fluorouracil (5-FU)/leucovorin (LV) in the locally advanced setting.

**Patients and methods:** This case-control study included 119 healthy controls and 97 patients with locally advanced rectal cancer (LARC). For *MTHFR* genotyping, restriction fragment length polymorphism analysis (PCR-RFLP) was employed.

**Results:** *In silico* analysis highlighted that SNPs C677T and A1298T correlate with *MTHFR* gene expression, and that gene expression profile correlates with cancer risk and stage. Using dominant and recessive models, it was found that the *MTHFR* 677CC vs. 677CT+677TT have increased risk of cancer development (odds ratio (OR): 2.27; 95% confidence interval (CI): 1.30–3.95, *p* = 0.002) as well as 677CC+677CT compared to 677TT (OR: 4.18, 95% CI: 1.16–14.99, *p* = 0.014). *MTHFR* 1298AA also shown increased risk for cancer development compared to 1298AC+1298CC (OR:2.0, 95% CI: 1.20–3.59, *p* = 0.035) Statistical analysis of combined genotypes highlighted the protective role of CT/AC combined genotype (OR: 3.15 95% CI: 1.576–6.279, *p* = 0.002) while the CC/AA genotype showed an increased risk for rectal cancer development (OR: 2.499, 95% CI: 1.246–5.081, *p* = 0.016) The carriers of the 677C/1298A haplotype had the highest risk for developing rectal cancer (OR: 1.74; 95% CI: 1.198–2.530, *p* = 0.002) while the 677T/1298C haplotype seems to provide a protective effect. (OR: 0.44; 95%CI 0.248–0.795, *p* = 0.003). No significant association with response to chemoradiotherapy was found.

**Conclusion:** Our data point to *MTHFR* 667C allele and 1298A alleles as low-penetrance risk factors for rectal cancer in our population. To the best of our knowledge, this is the first study of this type performed on the Slavic population in the Western Balkan, as various population-based factors might also be significant our findings can be used for future meta-analyses and the construction of genetic cancer risk prediction panels.

## 1 Introduction

Colorectal cancer (CRC) is the third most frequently diagnosed cancer with 1.93 million newly diagnosed patients in 2020, and the second leading cause of cancer death worldwide with 935 000 deaths annually (Sung et al., 2021). The situation in Serbia is similar to the global one, CRC is the second most frequent cancer with regards to both incidence and mortality, with around 5000 newly diagnosed CRC cases and around 2400 deaths in 2020 (Serbian Cancer Registry, 2022). It is a multifactorial disease involving both genetic and environmental factors. Approximately 75% of CRCs are sporadic and occur in cases of absence of genetic predisposition or family history of CRC (Yamagishi et al., 2016). Diet plays an important role in the development of colorectal malignancy, as well as smoking history, alcohol consumption, body mass index (BMI), and lifestyle, although the relative significance of each of these factors on its own, or combinations of more than one factor is not clear (Ryan-Harshman and Aldoori, 2007). Rectal and colon cancer are different diseases with regard to diagnosis, sensitivity to treatment, and some risk factors, but few studies addressed risk factors for these two cancers separately.

The role of folic acid deficiency has been investigated in tumorigenesis in general (Kim, 2003). Some of the proposed models of folic depletion influence on cancer development are alterations in DNA methylation, disruption of DNA integrity, and disruption of DNA repair (Choi and Mason, 2000). Interestingly, folate deficiency has been investigated as a factor only in alcohol-related carcinogenesis of rectal cancer, since there is a clear relationship between alcohol consumption and alterations in folate metabolism (Choi and Mason, 2000). Whether dietary intake of folate has a protective effect against selected cancers is not clear, since the results of studies are not consistent. An important protein in the metabolism of folic acid is the methylenetetrahydrofolate reductase (MTHFR). This enzyme converts the 5,10-methylenetetrahydrofolate to 5-methyltetrahydrofolate, the primary form of folate in blood.

Two common functional polymorphisms in the *MTHFR* gene are C677T (rs1801133; c.788C>T) and A1298C (rs1801131; c.1409A>C). These polymorphisms influence enzyme activity. The C677T polymorphism in exon 4 causes a substitution of C to T nucleotide, leading to the substitution of alanine with valine in codon 222, which in turn affects the active site of the enzyme and thus reduces its activity, with the TT genotype product having a 70% reduced activity in comparison to wild type. A substitution of A to C at nucleotide 1298 (polymorphism A1298C of the *MTHFR* gene) leads to the substitution of glutamine with alanine at the position 429, also causing reduced enzyme activity (Kennedy et al., 2012). The relationship between these polymorphisms and the risk of developing cancer has been observed and the results are contradictory and inconclusive. Some of the published studies showed differences in connection with ethnicity, as interestingly, there seems to be a higher frequency of the 677, TT genotype in southern Europe than in the north, while in Asia, the frequency is highest in China and lowest in India. Also, African Americans have a lower frequency of the TT genotype than Caucasians. The 1298CC genotype is more frequent in Caucasians (4%–12%) than in China and Japan (1%–4%) (Kennedy et al., 2012).

Preoperative, neoadjuvant chemoradiotherapy (nCRT) based on 5-fluorouracil (5-FU), followed in most cases by operative treatment is the standard of care for LARC (Glynne-Jones et al., 2017). Tumor regression grade (TRG) is an established prognostic factor for local recurrence, disease-free, and overall survival (OS), with significantly better outcomes in patients showing TRG 1–2 (good responders) than those with TRG 3–5 (poor responders) (Vecchio et al., 2005). The cytotoxic activity of 5-FU is exhibited mainly by its active metabolite which forms a complex with thymidylate synthase (TS) and 5,10-methylenetetrahydrofolate (5,10-MTHF) thus causing the inhibition of TS and disrupting normal DNA synthesis (Longley et al., 2003). Elevated intracellular 5,10-MTHF levels are needed for optimal inhibition of TS, and these are controlled by methylenetetrahydrofolate reductase (MTHFR), whose decreased activity results in higher levels of 5,10-MTHF and higher rate of inhibition of TS. There are large inter-individual differences in the efficacy of 5-FU. Considering the described mechanism of action of 5-FU, it is reasonable to assume that certain polymorphisms in genes involved in various points of 5-FU mechanism of action could explain some of these inter-individual differences in clinical response and toxicity to 5-FU (Ulrich et al., 2014). Polymorphisms in the MTHFR gene which cause decreased activity of MTHFR could make these patients more sensitive to 5-FU, therefore there should be a higher efficacy of 5-FU which in turn should lead to better survival. However, most study results do not support this hypothesis. Two polymorphisms of the *MTHFR* gene, C677T and A1298C, have been investigated in this context in patients with CRC and more extensively in patients with LARC treated with chemo-irradiation.

So far, various research that aimed to profile genetic risk factors of different types of cancer was conducted in Serbia to construct a general predictive risk model (Cavic et al., 2016; Krivokuca et al., 2016; Cavic et al., 2019). Results like these might contribute to the construction of a low-cost and minimally invasive pan-cancer polymorphism screening tool.

Most CRC cases in Serbia are discovered at an advanced stage, when there are few treatment choices and a low chance of survival. In order to improve overall patient care, our group and others have worked to profile the diagnostic, prognostic, and predictive characteristics for colorectal cancer (CRC), rectal and anal cancer (Cavic et al., 2016; Nikolic et al., 2021; Stojanovic- Rundic et al., 2021; Vuletić et al., 2021; Marinkovic et al., 2023; Stanojevic et al., 2023).

The aim of this study was to examine the association of *MTHFR* C677T and A1298C single nucleotide polymorphisms with the risk of rectal cancer as well as the response to neoadjuvant chemoradiotherapy based on 5-Fluorouracil (5-FU)/leucovorin (LV) in the locally advanced setting, in an effort to provide data from the Western Balkan area which is usually underrepresented in larger meta-analyses.

## 2 Materials and methods

### 2.1 *In silico* analysis using the Human Protein Atlas, UALCAN, ROCplotter, STRING, and NCBI GEOdatasets

The interactive online resource for examining cancer transcriptome data from the Cancer Genome Atlas (TCGA-ROAD) (Muzny et al., 2012; National Cancer Institute and the National Human Genome Research Institute, 2021) UALCAN (https://ualcan.path.uab.edu/analysis.html) (UALCAN database, n.d.; Chandrashekar et al., 2017) was employed to analyze *MTHFR* expression levels in normal and rectal cancer samples. The publicly available database the Human Protein Atlas (HPA) (https://www.proteinatlas.org) (The Human Protein Atlas database V.20.0, 2021; Uhlen et al., 2017) was used to analyze TCGA- ROAD transcriptome data on the expression of *MTHFR* in relation to its prognostic significance in rectal cancer (Muzny et al., 2012). Kaplan-Meier plots summarize the results of the correlation between *MTHFR* expression level and patient survival by assigning patients to groups that are either low (under experimental cut-off) or high (above experimental cut-off) groups. Expression cut-off values for the HPA data are presented as the number fragments per kilobase of exon per million reads (FPKM) of *MTHFR* in the tumor tissue at diagnosis. The transcriptome data of patients with rectal cancer were analyzed using an online tool ROCplotter (www.rocplot.org), to determine the *MTHFR* expression levels in responders and non-responders (Fekete and Győrffy, 2019). Corresponding images and data were downloaded from the HPA, UALCAN and ROCplotter platforms in the original form. The STRING (https://string-db.org, Szklarczyk et al., 2022) protein network for MTHFR was built based on the highest confidence (0.9) evidence from experimental and biochemical data, co-expression, gene neighborhood, gene co-occurrence, gene fusions, protein homology, manually curated metabolic and signaling pathway databases, and predictive and knowledge text data mining. The network included 5 primary-interaction shell proteins to explore interactions and clustering with other proteins and the effects of these interactions. For the enrichment analysis, the whole genome statistical background was assumed. The analysis was performed using STRING v.11.0 (Szklarczyk et al., 2022), corresponding images and results were exported and statistical significance was considered for *p* < 0.05. Colonomics web-based tool (https://colonomics.org) was used to access expression quantitative trait loci (eQTL) analysis for *MTHFR* gene expression depending on SNP C677T and A1298C (Moreno et al., 2018).

Gene Expression Omnibus (GEO) database (Barrett et al., 2012) was used for the search of publicly available datasets for external validation of obtained results. Search criteria included the keyword “rectal cancer” while “genome variation profiling by SNP array” and “SNP genotyping by SNP array” were used as a study type of interest.

### 2.2 Patients and controls

A case-control study was performed in a group of 97 patients diagnosed with locally advanced primary rectal adenocarcinoma (age range 29–83 years, median 61; 67 males, 30 females) from several cancer centers in Serbia treated at the Institute for Oncology and Radiology of Serbia from 2018–2019, and 119 healthy control subjects (age range 32–89 years, median 55; 67 males, 52 females) with no previous history of malignancies and no known folate metabolism deficiency, all of Caucasian descent (Table 1). In adherence to the National guidelines for colorectal cancer screening, colonoscopies were conducted on individuals in the control group when there was an indication based on these criteria. Importantly, the results of these colonoscopies were negative for colorectal cancer at the time of examination. All patients were diagnosed with locally advanced rectal cancer, stage II (T3/4N0M0) or III (T1-4N + M0) according to clinical and histological criteria of the 8^th^ edition of the TNM classification of malignant tumors, and ECOG ≤ 2 (Oken et al., 1982). The tumors were located <15cm from the anocutaneous line and were treated with neoadjuvant chemoradiotherapy (5-Fluorouracil 350 mg/m2 i. v. bolus plus Leucovorin 25mg/m2 D1-D5 and D29-D33). Radiotherapy was initiated concurrently with chemotherapy, 50.4 Gy in 28 fractions, conventionally fractioned 1.8Gy/fr, using the technique with 3 or 4 radiation areas (all areas as endorsed by the International Committee of Radiation Units and Measurements (ICRU) 50/62) (Landberg et al., 2016a; Landberg et al., 2016b). Clinical response assessment took place 6–8 weeks after the completion of neoadjuvant therapy, involving pelvic MRI scans, rigid proctoscopy, and digital rectal examinations. Subsequently, patients were referred for surgery. The patohistological assessment of surgical specimens included the determination of histomorphology of the resected tumor (type and grade), tumor invasiveness (ypTNM, R classification) (Wei et al., 2018), pathohistological grading of the tumor regression by the Mandard scale (Siddiqui et al., 2016) with the determination of prognostic categories.

**TABLE 1:** Patient characteristics.

Characteristic	Patients N (%)
**Age (years)**	
Mean (SD)	59.9 (9.9)
Median (Range)	61.0 (38–76)
**Gender**	
Female	30 (30.9)
Male	67 (69.1.7)
**UICC staging** (Union for International Cancer Control, 2020)	
II	8 (8.2)
III	89 (91.8.2)
**Grade**	
1	63 (63.8)
2	28 (30.4)
3	4(4)
NA	2 (2)
**RT technique**	
2D	2 (2)
3D	93(98)
NA	2
**RT dose (Gy)**	
Mean (SD)	48.8 (2.9)
Median (Range)	50.4 (36–50.4)
**Tumor regression grade (TRG)** ^a^	
1	15 (15.5)
2	15 (15.5)
3	28 (28.9)
4	35 (36.1)
5	2 (2.1)
NA	2(2.1)

^a^ According to Mandard scale(Siddiqui et al., 2016), NA, data unavailable, SD, standard deviation, UICC (Union for International Cancer Control, 2020), the union for international cancer control, RT, radiotherapy.

To investigate the predictive role of *MTHFR* polymorphisms, patients were divided into responders (patients with tumor regression grades (TRG 1 and 2)) and, non-responders (TRG 3–5) based on postoperative specimens. Two out of 97 patients enrolled in the study didn’t have TRG status at the moment of analysis. Patients who achieved a complete clinical response without subsequent operative treatments were excluded from this analysis.

### 2.3 DNA isolation and *MTHFR* genotyping

Genomic DNA was isolated from formalin-fixed paraffin-embedded tissue samples (FFPE) obtained by biopsy/resection using the QIAamp^®^ DNA FFPE Tissue isolation kit (Qiagen, United Kingdom). Ethylenediaminetetraacetic acid (EDTA) peripheral blood was drawn from healthy controls by venipuncture and further used for leukocyte isolation using BloodPrep Chemistry for ABI PRISM™ 6100 Nucleic Acid PrepStation (Applied Biosystems, CA, United States). The concordance between germline and somatic DNA in variations of pharmacogenetic genes is around 100%, according to a recent large-scale literature research. Spectrophotometric analysis was used to evaluate the concentration and purity of the extracted DNA samples (Nanodrop, Shimadzu). For *MTHFR* genotyping, restriction fragment length polymorphism analysis (PCR-RFLP) was employed as previously described (Jakovljevic et al., 2012; Cavic et al., 2014; 2016). The analysis was performed by gel electrophoresis on a chip using the Agilent DNA 1000 Kit on the Agilent 2100 Bioanalyzer. To ensure adequate genotyping, a previously established heterozygote sample was used as a method of control, and genotyping was carried out blind to case-control status. 10% of samples, chosen at random, underwent Sanger sequencing analysis to verify the accuracy of the findings.

### 2.4 Statistical analyses

The sample data were summarized using descriptive statistical methods (frequencies, percentages, means, medians, standard deviation, SD, and range). The Hardy-Weinberg equilibrium of the analyzed polymorphisms was tested using the Pearson Chi-Square test. Two-sided *p* values < 0.05 were considered to indicate statistical significance. The associations between the patients’ and healthy controls as well as responders and Non-responders were analyzed by applying Pearson Chi-Square with Yates’ correction. Fisher’s exact test was used to analyze differences between males and females. Combined genotype frequencies were calculated by direct counting while statistical significance in combined genotype distribution between patients and controls was observed using the Chi-Square test with Yates’ correction. In addition, haplotype analysis was used for calculating the interaction between two polymorphic sites of *MTHFR*. Haplotype frequencies were calculated manually, and data were confirmed using Multiallelic Interallelic Disequilibrium Analysis Software (University of Southampton, Highfield, Southampton, United Kingdom) (Gaunt et al., 2006) and Golden Helix Tree SNP and Variation Suite software (Golden Helix, Bozeman, MT, United States). Statistical significance was obtained using Pearson Chi-Square with Yates’ correction. All statistical analysis was performed using GraphPad Prism 8.0.1(GraphPad Software, Boston, MA, United States) and SNPstat (https://www.snpstats.net/start.html).

## 3 Results

### 3.1 *In silico* analyses

**FIGURE 1:**
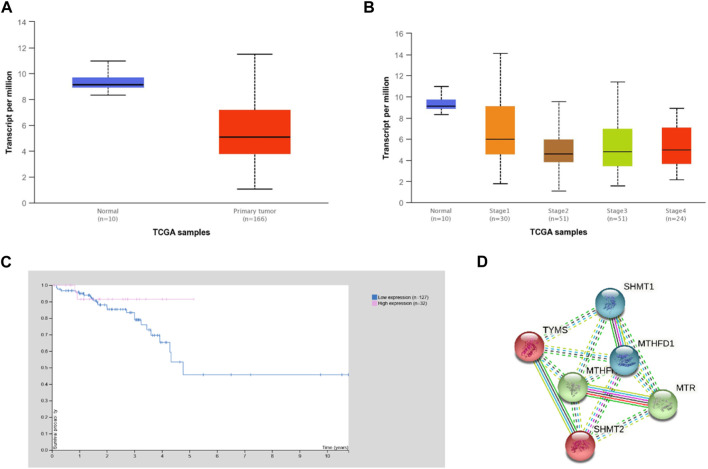
Expression of *MTHFR* in rectal cancer according to the UALCAN and HPA analysis of TCGA data. **(A)** Expression of *MTHFR* in rectal adenocarcinoma based on sample type (normal vs. cancer tissue *p* = 0.013). **(B)** Expression of *MTHFR* in rectal adenocarcinoma by stages compared to normal tissue (normal vs. stage 2, *p* = 0.003; normal vs. stage 3; *p* = 0.001; normal vs. stage 4, *p* = 0.001) and between individual cancer stage (stage 1 vs. stage 3, *p* = 0.020; stage 1 vs. stage 4, *p* = 0.041). **(C)** Survival curves of rectal cancer patients in relation to the expression of *MTHFR* (expression cut-off 3.14 FPKM, *p* = 0.270). **(D)** Direct STRING (STRING database, n.d.) network of MTHFR was built based on highest confidence (0.9) evidence from experimental interaction data (pink), co-expression (black), gene neighborhood (green) and co-occurrence (blue) data, curated databases (light blue), predictive and knowledge text mining (light green), protein homology (purple). The network included 5 primary-interaction shell genes to explore their indirect interactions and clustering on all analyzed platforms (PPI enrichment *p*-value: 0.002). Red nodes–TYMS cluster members; green nodes–MTHFR cluster members; blue nodes–MTHFD1 cluster. Nodes are labeled with HGNC symbols: MTHFD1, methylenetetrahydrofolate dehydrogenase, cyclohydrolase and formyltetrahydrofolate synthetase 1 MTHFR - methylenetetrahydrofolate reductase; MTR, methionine synthase; TYMS, thymidylate synthase; SHMT1, serine hydroxymethyltransferase, cytosolic; SHMT2, serine hydroxymethyltransferase, mitochondrial.

*In silico* analyses using the UALCAN and HPA platforms showed that *MTHFR* expression was significantly higher in normal compared to rectal cancer tissue (Figure 1A, *p* = 0.013). Expression of *MTHFR* in rectal adenocarcinoma by stages 2 and 3 compared to normal tissue showed a statistical significant difference (normal vs. stage 2, *p* = 0.003; normal vs. stage 3; *p* = 0.001; normal vs. stage 4, *p* = 0.001) and low expression correlated with higher cancer stages (Figure 1B; stage 1 vs. stage 3, *p* = 0.020; stage 1 vs. stage 4, *p* = 0.050). It was generally not prognostically significant in rectal cancer (*p* = 0.270), but the 5-year survival rate was found to be 91% for the high expression group and 48% for the low expression group (Figure 1C; expression cut-off 3.14 FPKM, median follow up time 1.75 years).

ROC analysis included 42 patients in total (19 responders and 23 non-responders) and highlighted that *MTHFR* expression (Affy ID 7436) is slightly higher in a group of patients who responded poorly to the therapy but without statistical significance. There was weak statistical significance in ROC *p*-value (*p* = 0.045) with area under the curve (AUC = 0.648).

The STRING analysis showed that *MTHFR* has direct and indirect interactions with various proteins that are important for rectal cancerogenesis/homeostasis when gene co-expression, experimental/biochemical data, and text mining were considered at the highest confidence level (0.9) (Figure 1D). Cluster analysis extended to 5 primary-interaction shell genes (*MTHFD1* - methylenetetrahydrofolate dehydrogenase, cyclohydrolase and formyltetrahydrofolate synthetase 1, *MTR* - methionine synthase, *TYMS* - thymidylate synthase, *SHMT1* - serine hydroxymethyltransferase, cytosolic and *SHMT2* - serine hydroxymethyltransferase, mitochondrial) showed that these proteins form biological clusters across all analyzed platforms. The extended network was found to be enriched in interactions (protein-protein interactions PPI enrichment *p*-value: 0.002), which indicated that they interact with each other significantly more than is expected for a random set of proteins of similar size and can be thus considered as a biologically interconnected group (Szklarczyk et al., 2012).

**FIGURE 2:**
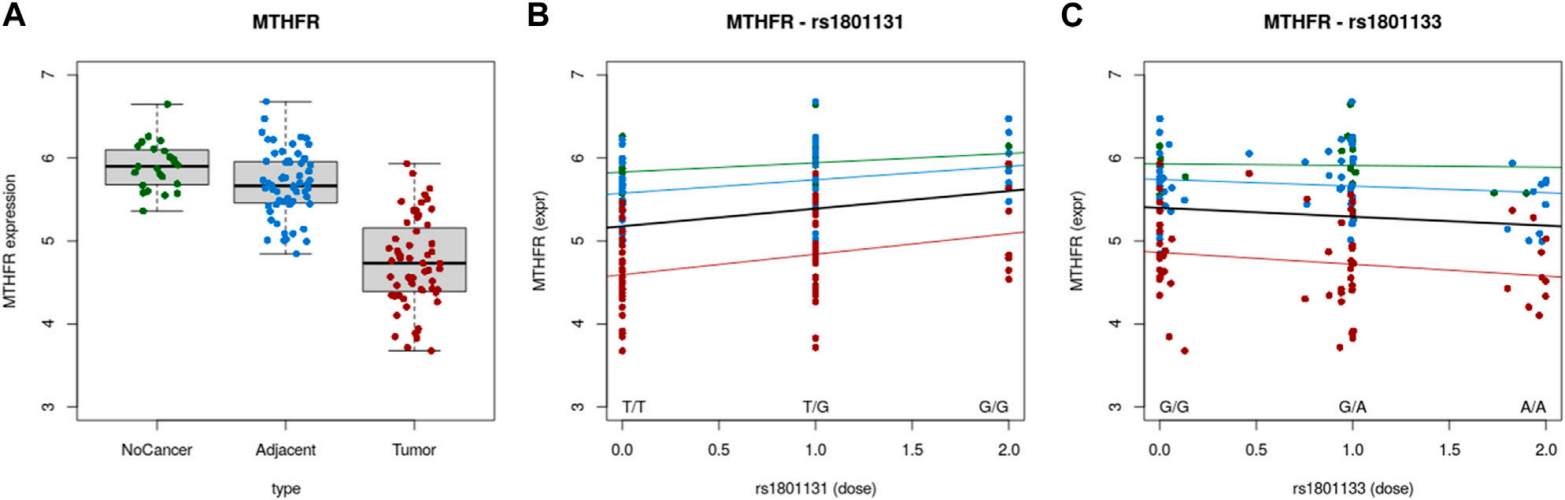
**(A)** Expression of *MTHFR* in normal, adjacent and tumor tissue. **(B)** Correlation between *MTHFR* expression and A1298C polymorphic variants. **(C)** Correlation between *MTHFR* expression and C677T polymorphic variants.

Correlation between gene expression and SNPs were evaluated using Colonomics online tool (https://www.colonomics.org/data-browser/dashboard/). eQTL analysis was performed for left colon samples (normal, adjacent and tumor tissue) for both SNPs (C677T and A1298C). As rectal tissue data were not available, left colon was analyzed as anatomically closest to rectal tissue thus expecting similarity in expression profiles. This was confirmed by comparing the *MTHFR* expression profile of the left colon (Figure 2A) and rectal adenocarcinoma (Figure 1B) *MTHFR* gene expression of 138 individuals (Normal N = 20; Adjacent N = 59 and Tumor N = 59) confirmed that tumor tissue had overall lower *MTHFR* expression compared to adjacent and normal tissue, respectively. eQTL analysis highlighted a clear correlation between gene expression and SNPs in both cases. The existence of mutation A>C at 1298 position, positively affected *MTHFR* expression in every type of tissue (Figure 2B), while C>T at position 677 had an effect of a decrease of *MTHFR* expression for each type of tissue (Figure 2C).

### 3.2 *MTHFR* genotyping

**FIGURE 3:**
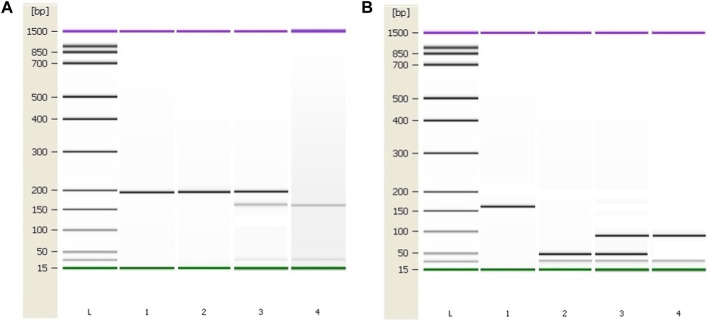
Genotyping results of the *MTHFR* C677T and A1298C polymorphic variants using the Agilent 2100 Bioanalyzer. **(A)** PCR and RFLP results of the *MTHFR* C677T polymorphic variant. Column 1: 198 bp PCR product. Column 2: C/C, Column 3: C/T, Column 4: T/T **(B)** PCR and RFLP genotyping results of the *MTHFR* A1298C polymorphic variant. Column 1: 163 bp PCR product. Column 2: A/A, Column 3: A/C, Column 4: C/C. L–High-sensitivity DNA ladder (Agilent Technologies). 1500 bp upper and 15 bp lower marker are present in each column.

Patient characteristics are presented in Table 1. PCR was used to amplify regions of interest using predesigned primers for *MTHFR* C677T region (forward 5′ TGA AGG AGA AGG TGT CTG CGG GA 3′ and reverse 5′ AGG ACG GTG CGG TGA GAG TG 3′) and for *MTHFR* A1289C region (forward 5′ CTT TGC CAT GTC CAC AGC ATG 3′ and reverse 5′ AAG GAG GAG CTG CTG AAG ATG 3’). From all patient and control samples, a 198 bp PCR product containing the MTHFR C677T polymorphism site was produced with success (Figure 3A). Digestion was performed using Fast Digest HinfI and Fast Digest MboII enzymes, respectively (Thermo Fisher Scientific, Waltham, Massachusetts, United States). After digestion of the PCR products, homozygotes (CT) formed three bands of 198, 175, and 23 bp, and homozygotes (TT) produced two fragments of 175 and 23 bp. An undigested PCR product (198 bp) showed the presence of a homozygous wild-type genotype (CC). From each patient and control sample, a 163 bp PCR product containing the MTHFR A1298C polymorphism site was produced with success (Figure 3B). Four bands measuring 84, 31, 30, and 18 bp occur when the homozygous wild type A allele is present, while five bands measuring 56, 31, 30, 28, and 18 bp appear when the C allele is present.

### 3.3 Significance for cancer risk

**FIGURE 4:**
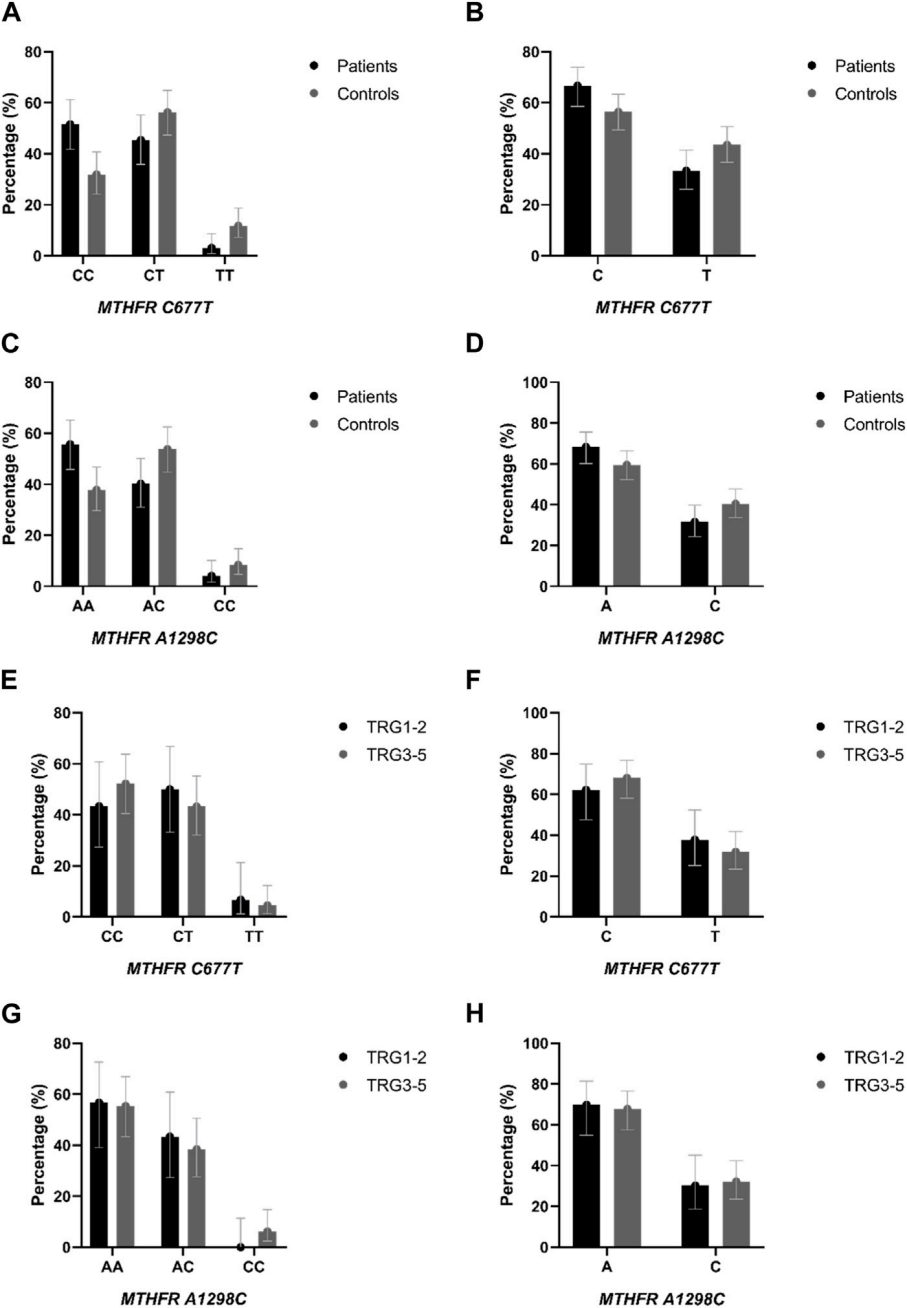
**(A)** Genotype and **(B)** allele distribution of the *MTHFR* C677T polymorphic variants in rectal cancer patients and healthy controls. **(C)** Genotype and **(D)** allele distribution of the *MTHFR* A1298C polymorphic variants in rectal cancer patients and healthy controls. **(E)** Genotype and **(F)** allele distribution of the *MTHFR* C677T polymorphic variants in responders and non-responders to neoadjuvant chemoradiotherapy. **(G)** Genotype and **(H)** allele distribution of the *MTHFR* A1298C polymorphic variants in responders and non-responders to neoadjuvant chemoradiotherapy.

Distribution of genotypes, *MTHFR* C677T and *MTHFR* A1298C distribution in patients and controls did not deviate from the Hardy-Weinberg equilibrium. The allele frequencies of the *MTHFR* C677T polymorphic variants in patients and controls revealed that patients had a higher frequency of C allele (C = 0.74; T = 0.26) than healthy controls (C = 0.60; T = 0.40) with trend in statistical significance *p* = 0.050 (Table 2; Figures 4A, B). Analysis of the effects of *MTHFR* C677T and A1298C polymorphic variants on rectal cancer risk using codominant, dominant, recessive and overdominant models was represented using *p* value < 0.05; odds ratio (OR) and 95% confidence interval which are shown in Table 3, using a previously published method (Ozretić et al., 2019). The codominant model shown statistical significance between prevalence of 677CC, 677CT and 677TT within patient and control group (*p* = 0.003). Using dominant and recessive models, it was found that the *MTHFR* 677CC vs. 677CT+677TT have increased risk of cancer development (OR: 2.27; 95% CI: 1.30–3.95, *p* = 0.002) as well as 677CC+677CT compared to 677TT (OR: 4.18, 95% CI: 1.16–14.99, *p* = 0.014). Using codominant (*p* = 0.013), dominant and recessive models, we found association with cancer risk in group of males. Using recessive models, it was found that 677TT has a protective effect against rectal cancer development (OR: 5.70; 95% CI: 1.20–27.11; *p* = 0.012). In the female group, using the dominant model it was found that 677TT has a protective effect (OR: 2.57; 95% CI: 1.02–6.50; *p* = 0.044).

**TABLE 2:** Genotype and allele frequencies of *MTHFR* C677T and A1298C polymorphic variants in the patient and healthy control groups.

Genotype	N (%)	Allele frequency
*MTHFR* C677T		
**Patients**		
CC	50 (51.5.)	C 0.74
CT	44 (45.4)	T 0.26
TT	3 (3.1)	
**Controls**		
CC	38 (31.9)	C 0.60
CT	67 (56.3)	T 0.40
TT	14 (11.8)	
**Responders (TRG1-2)**		
CC	13 (43.3)	C 0.68
CT	15 (50.0)	T 0.32
TT	2 (6.7)	
**Non-responders (TRG3-5)**		
CC	35 (53.8)	C 0.76
CT	29 (44.6)	T 0.24
TT	1 (1.6)	
*MTHFR* A1298C		
**Patients**		
AA	54 (55.7)	A 0.76
AC	39 (40.2)	C 0.24
CC	4 (4.1)	
**Controls**		
AA	45 (37.8)	A 0.65
AC	64 (53.8)	C 0.35
CC	10 (8.4)	
**Responders (TRG1-2)**		
AA	17 (56.7)	A 0.78
AC	13 (43.3)	C 0.22
CC	0 (0.0)	
**Non-responders (TRG3-5)**		
AA	36 (55.4)	A 0.75
AC	25 (38.5)	C 0.25
CC	4 (6.1)	

* Values < 3.841 are not significant at *α* = 0.05(Nielsen and Slatkin, 2013), TRG-tumor regression grade according to the Mandard scale.

**TABLE 3:** Analysis of the effects of *MTHFR* C677T and A1298C polymorphic variants on rectal cancer risk using codominant, dominant, recessive and overdominant models.

SNP		Genotype	N (%) patients	N(%) controls	OR (95% CI)	*p* value
*MTHFR* C677T	Codominant	CC	50 (51.5)	38 (31.9)	1	**0.003**
		CT	44 (45.4)	67 (56.3)	2.00 (1.14–3.54)	
		TT	3 (3.1)	14 (11.8)	6.14 (1.65–22.60)	
	Dominant	CC	50 (51.5)	38 (31.9)	1	**0.003**
		CT + TT	47 (48.5)	81 (68.1)	2.27 (1.30–3.95)	
	Recessive	CC + CT	94 (96.9)	105 (88.2)	1	**0.014**
		TT	3 (3.1)	14 (11.8)	4.18 (1.16–14.99)	
	Overdominant	CC + TT	53 (54.6)	52 (43.7)	1.00	0.110
		CT	44 (45.4)	67 (56.3)	1.55 (0.90–2.66)	
*MTHFR* C677T Males	Codominant	CC	34 (50.8)	22 (32.8)	1.00	**0.013**
		CT	31 (46.3)	35 (52.2)	1.74 (0.85–3.59)	
		TT	2 (3.0)	10 (14.9)	7.73 (1.54–38.66)	
	Dominant	CC	34 (50.8)	22 (32.8)	1.00	**0.035**
		CT + TT	33 (49.2)	45 (67.2)	2.11 (1.05–4.24)	
	Recessive	CC + CT	65 (97)	57 (85.1)	1.00	**0.012**
		TT	2 (3.0)	10 (14.9)	5.70 (1.20–27.11)	
	Overdominant	CC + TT	36 (53.7)	32 (47.8)	1.00	0.490
		CT	31 (46.3)	35 (52.2)	1.27 (0.64–2.50)	
*MTHFR* C677T Females	Codominant	CC	16 (53.5)	16 (30.8)	1.00	
		CT	13 (43.3)	32 (61.5)	2.46 (0.96–6.34)	0.120
		TT	1 (3.3)	4 (7.7)	4.00 (0.40–39.83)	
	Dominant	CC	16 (53.3)	16 (30.8)	1.00	**0.044**
		CT + TT	14 (46.7)	36 (69.2)	2.57 (1.02–6.50)	
	Recessive	CC + CT	29 (96.7)	48 (92.3)	1.00	0.410
		TT	1 (3.3)	4 (7.7)	2.42 (0.26–22.68)	
	Overdominant	CC + TT	17 (56.7)	20 (38.5)	1.00	0.110
		CT	13 (61.5)	32 (61.5)	2.09 (0.84–5.21)	
*MTHFR* A1298C	Codominant	AA	54 (55.7)	45 (37.8)	1.00	**0.025**
		AC	39 (40.2)	64 (53.8)	1.97 (1.12–3.45)	
		CC	4 (4.1)	10 (8.4)	3.00 (0.88–10.21)	
	Dominant	AA	54 (55.7)	45 (37.8)	1.00	**0.009**
		AC + CC	43 (44.3)	74 (62.2)	2.07 (1.20–3.56)	
	Recessive	AA + AC	93 (95.9)	109 (91.6)	1.00	0.190
		CC	4 (4.1)	10 (8.4)	2.13 (0.65–7.03)	
	Overdominant	AA + CC	58 (59.8)	55 (46.2)	1.00	**0.046**
		AC	39 (40.2)	64 (53.8)	1.73 (1.01–2.98)	
*MTHFR* A1298C Males	Codominant	AA	39 (58.2)	28 (41.8)	1.00	0.140
		AC	24 (35.8)	35 (52.2)	2.03 (1.00–4.14)	
		CC	4 (6.0)	4 (6.0)	1.39 (0.32–6.05)	
	Dominant	AA	39 (58.2)	28 (41.8)	1.00	0.057
		AC + CC	28 (41.8)	39 (58.2)	1.94 (0.98–3.85)	
	Recessive	AA + AC	63 (94.0)	63 (94.0)	1.00	1.000
		CC	4 (6.0)	4 (6.0)	1.00 (0.24–4.18)	
	Overdominant	AA + CC	43 (64.2)	32 (47.8)	1.00	0.055
		AC	24 (35.8)	35 (52.2)	1.96 (0.98–3.92)	
*MTHFR* A1298C Females	Codominant	AA	15 (50.0)	17 (32.7)	1.00	**0.030**
		AC	15 (50.0)	29 (55.8)	1.71 (0.67–4.34)	
		CC	0 (0.0)	6 (11.5)	NA	
	Dominant	AA	15 (50.0)	17 (32.7)	1.00	0.120
		AC + CC	15 (50.0)	35 (67.3)	2.06 (0.82–5.17)	
	Recessive	AA + AC	30 (100.0)	46 (88.5)	1.00	**0.017**
		CC	0 (0.0)	6 (11.5)	NA	
	Overdominant	AA + CC	15 (50.0)	23 (44.2)	1.00	0.610
		AC	15 (50.0)	29 (55.8)	1.26 (0.51–3.10)	

OR, odds ratio; CI, confidence interval; Statistically significant results are bolded.

The frequency of the A allele was slightly higher in patients (A = 0.76; C = 0.24) than in healthy controls (A = 0.65; C = 0.35) (*p* = 0.121) (Table 2; Figure 4D). AA homozygosity at the 1298 polymorphic site of the *MTFHR* gene was associated with a higher risk of developing rectal cancer in the dominant model (Table 3; Figure 4C). *MTHFR* 1298AA also showed increased risk for cancer development compared to 1298AC+1298CC (OR: 2.07, 95% CI: 1.20–3.56, *p* = 0.009). It was also found that the overdominant model predicted a protective effect of 1298AC compared to 1298AA and 1298CC (OR: 1.73; 95% CI: 1.01–2.98; *p* = 0.046). The recessive model in the female population showed that A1298CC had a protective effect for rectal cancer development (*p* = 0.017). There were no differences in male population with regard to the effect of this polymorphism and risk for rectal cancer.

### 3.4 Combined genotype

In groups of patients and controls, the 4 common combined genotypes were CT/AA (29.90% vs. 19.33%), CC/AC (24.74% vs. 15.97%), CC/AA (24.74% vs. 12.61%), and CT/AC (13.40% vs. 32.77%) (Table 4). Statistical analysis of combined genotypes highlighted the protective role of CT/AC combined genotype (*p* = 0.002) while the CC/AA genotype showed an increased risk for rectal cancer development (*p* = 0.016) (Table 5).

**TABLE 4:** Combined genotype frequencies of *MTHFR* C677T and A1298C polymorphic variants within the patients and controls.

Combined genotype	N (%) patients	N (%) R	N(%) NR	N (%) controls
**CT/AA**	**29 (29.9)**	**10 (33.3)**	**19 (29.2)**	**23 (19.3)**
**CC/AC**	**24 (24.7)**	**6 (20.0)**	**17 (26.1)**	**19 (16)**
**CC/AA**	**24 (24.7)**	**7 (23.3)**	**16 (24.6)**	**15 (12.6)**
**CT/AC**	**13 (13.4)**	**5 (16.7)**	**8 (12.3)**	**39 (32.8)**
TT/AC	2 (2.1)	2 (6.7)	0 (0)	6 (5.0)
CT/CC	2 (2.1)	0 (0)	2 (3.1)	5 (4.2)
CC/CC	2 (2.1)	0 (0)	2 (3.1)	4 (3.4)
TT/AA	1 (1.0)	0 (0)	1 (1.5)	7 (5.9)
TT/CC	0 (0.0)	0 (0)	0 (0)	1 (0.8)

Combined genotypes with the highest prevalence are labeled bold.

**TABLE 5:** The effects of *MTHFR* C677T and A1298C combined genotype on rectal cancer risk.

Combined genotype	Patients vs. Healthy controls OR (95% CI)	*p* value (Pearson χ^2^ test)
CC/AA vs. any other	2.499 (1.246–5.081)	**0.016**
CT/AC vs. any other	3.15 (1.576–6.279)	**0.002**

Statistically significant results are labeled bold, OR, odds, ratio, CI, confidence interval.

### 3.5 Haplotype analysis

Haplotype analysis indicated that the most frequent haplotypes in patients vs. controls were CA (677C-1298A) (46.15% vs. 32.99%) and TA (677T-1298A) (24.10% vs. 26.04%) followed by CC (677C–1298C) (21.03% vs. 23.26%). The rarest haplotype was TC (677T-1298C) (8.72% vs. 17.71%). The carriers of the CA haplotype had the highest risk for developing rectal cancer ((OR): 1.74; 95% (CI) 1.198–2.530, *p* = 0.002) while the TC haplotype seems to provide a protective effect. (OR: 0.44; 95%CI 0.248–0.795, *p* = 0.003). These results indicate that the two loci 677 and 1298 share relatively weak linkage disequilibrium in the patient group represented by coefficient of linkage disequilibrium (D′) and correlation coefficient (*r* ^2^) (D` = 0.27, *r* ^2^ = 0.00797) (Table 6).

**TABLE 6:** Estimated haplotype frequencies of *MTHFR* C677T and A1298C polymorphisms in patients and controls.

Haplotype	N (%) patients	N (%) controls	OR (95% CI)	*p* value
677C-1298A*	90 (46.15)	95 (32.99)	1.74 (1.190–2.510)	**0.005**
677C–1298C	41 (21.03)	67 (23.26)	NA	0.537
677T-1298A	47 (24.10)	75 (26.04)	NA	0.339
677T-1298C	17 (8.27)	51 (17.71)	2.25 (1.286–3.977)	**0.008**

Statistically significant results are labeled bold; OR, odds, ratio; CI, confidence interval, NA-not, applicable; *reference haplotype labeled with asterisk.

### 3.6 Significance for response to chemoradiotherapy

Genotypes *MTHFR* C677T and *MTHFR* A1298C distribution in responders and non-responders did not deviate from the Hardy-Weinberg equilibrium. The allele frequencies of the *MTHFR* C677T polymorphic variants in responders and non-responders revealed that responders had a lower frequency of C allele (C = 0.68; T = 0.32) than non-responders (C = 0.76; T = 0.24) (*p* = 0.270) (Table 2; Figures 4E, F) but no statistical significance was obtained.

The frequency of the A allele in *MTHFR* A1298C genotype was lower in responders (A = 0.78; C = 0.22) than in non-responders (A = 0.75; C = 0.25) (Table 2; Figures 4G, H) but no statistical significance in predictive potential was obtained (*p* = 0.739). Analysis of the effects of MTHFR C677T and A1298C polymorphic variants on response to neoadjuvant chemoradiotherapy using codominant, dominant, recessive and overdominant models did not meet statistical significance (Table 7). Within the groups of responders and non-responders, no statistical significance was obtained in terms of predictive potential of combined genotypes as well as haplotype frequencies. Haplotype analysis indicated that the two loci 677 and 1298 show relatively strong linkage disequilibrium in the non-responder group (D` = 0.46, *r* ^2^ = 0.02163).

**TABLE 7:** Analysis of the effects of *MTHFR* C677T and A1298C polymorphic variants on response to neoadjuvant chemoradiotherapy using codominant, dominant, recessive and overdominant models.

SNP		Genotype	Responders	Nonresponders	OR (95% CI)	*p* value
*MTHFR* C677T	Codominant	CC	13 (43.3)	35 (53.9)	1.00	0.340
		CT	15 (50.0)	29 (44.6)	1.39 (0.57–3.39)	
		TT	2 (6.7)	1 (1.5)	5.38 (0.45–64.52)	
	Dominant	CC	13 (43.3)	35 (53.9)	1.00	0.340
		CT + TT	17 (56.7)	30 (46.1)	1.53 (0.64–3.65)	
	Recessive	CC + CT	28 (93.3)	64 (98.5)	1.00	0.200
		TT	2 (6.7)	1 (1.5)	4.57 (0.40–52.51)	
	Overdominant	CC + TT	15 (50.0)	36 (55.4)	1.00	0.620
		CT	15 (50.0)	29 (44.6)	1.24 (0.52–2.95)	
*MTHFR* A1298C	Codominant	AA	17 (56.7)	36 (55.4)	1.00	0.210
		AC	13 (43.3)	25 (38.5)	1.10 (0.45–2.67)	
		CC	0 (0.0)	4 (6.2)	NA	
	Dominant	AA	17 (56.6)	36 (55.4)	1.00	0.910
		AC + CC	13 (43.3)	29 (44.6)	0.95 (0.40–2.27)	
	Recessive	AA + AC	30 (100.0)	61 (93.8)	1.00	0.078
		CC	0 (0.0)	4 (6.2)	NA	
	Overdominant	AA + AA	17 (56.7)	40 (61.5)	1.00	0.650
		AC	13 (43.3)	25 (38.5)	1.22 (0.51–2.94)	

OR- odds ratio; CI- confidence interval.

### 3.7 Validation of results

Seven studies were found, but after initial processing one study, GSE35282 met all criteria and included polymorphic variant A1298C data of *MTHFR* from 43 patients with diagnosed locally advanced rectal cancer (Kim et al., 2013). There was no study available with the C677T polymorphic variant of *MTHFR*. Patients were divided according to Mandard TRG status into responders (TRG1/2; 41.9%) and non-responders (TR3/4; 58.1%, there were no patients with TRG5). Data obtained in the mentioned study showed high association with our results. Statistical significance was not found within the predictive potential of the A1298C *MTHFR* polymorphic variant while allele frequencies showed a high association between these two studies (Table 8).

**TABLE 8:** External validation of obtained results using publicly available dataset GSE35282.

A1298C polymorphic site *MTHFR*	Allele frequency GSE35282	Allele frequency our results	*p* value Pearson χ^2^ test
Patients	A 0.84	A 0.76	0.158
	C 0.13	C 0.24	
Responders	A 0.77	A 0.78	0.370
	C 0.23	C 0.22	
Non-responders	A 0.84	A 0.76	0.094
	C 0.16	C 0.24	

## 4 Discussion

Since colorectal cancer remains one of the most common and deadly malignant diseases worldwide, much effort is directed towards elucidating the etiopathological mechanisms and risk factors. Some risk factors are well known such as diet, alcohol consumption, smoking history and lifestyle but they alone are not enough to explain the development of the disease in all patients.

*In silico* analysis showed that there was a difference in the expression of normal and tumor tissue. In addition, higher stage of the disease was associated with a lower expression of the MTHFR gene. eQTL analysis indicated that the variant correlated positively (A1298C) and negatively (C677T) with the level of gene expression independent of the tissue type. These results were the basis for examining the predictive potential of *MTHFR* SNP when it comes to the response to neoadjuvant chemoradiotherapy.

Folate metabolism, more precisely folate deficiency is a very well-known factor of tumorigenesis in general since it plays an important role in maintaining genomic stability by being involved in DNA synthesis, repair, and methylation (Levin and Varga, 2016). 5,10-methylenetetrahydrofolate reductase (MTHFR), one of the key enzymes in the metabolism of folate, is responsible for the irreversible conversion of 5,10-methylenetetrahydrofolate to 5-methyltetrahydrofolate, which supplies a methyl group for the production of methionine, the lack of which can interfere with DNA synthesis (Ryan and Weir, 2001). Two common functional polymorphisms in the *MTHFR* gene that have been found to influence the risk of CRC are C677T and A1298C. These SNPs cause reduced activity of the MTHFR enzyme, thus influencing folate metabolism, causing folate depletion and DNA hypomethylation, and disruption of DNA synthesis and repair (Kennedy et al., 2012). DNA hypomethylation is a nearly universal early event in carcinogenesis. It has been suggested that site-specific DNA hypomethylation may be critical, such as the hypomethylation of the coding region (exons 5–8) of the p53 tumor suppressor gene, which is the most frequently mutated in human cancers (Kim et al., 1997; Choi and Mason, 2000).

Data regarding the specific influence of these polymorphisms on CRC risk are varied and inconclusive, and importantly, very few studies investigated the effect of these polymorphisms on the risk of rectal and colon cancer separately, which could be different since rectal and colon cancer are different diseases in their pathogenesis, histology, and sensitivity to treatment. We aimed to investigate the effect of C677T and A1298C polymorphisms on the risk of developing rectal cancer only, in the population of patients in Serbia. The results of this case-control study on 97 patients diagnosed with LARC and 119 healthy volunteers showed a higher frequency of the C677T C allele in patients than in controls (0.74), and the CC homozygosity at the 677 polymorphic sites was more common in patients than in healthy controls. This would suggest a protective effect of the T allele against rectal cancer. These results were confirmed using codominant, dominant, recessive and overdominant model of association. Data from previous studies are inconclusive, with most studies being carried out on single, ethnically homogenous populations, as is ours. Murtaugh et al. in the United States (Murtaugh et al., 2007), Levine et al. in Canada (Levin and Varga, 2016), Sheng et al. (Sheng et al., 2012) and Rai (Rai, 2016) in Asia showed the protective effect of the 677 T allele against CRC and rectal cancer in the American study, as in the situation in our study. In a meta-analysis of 67 studies carried out in 25 countries over the world and comprising all ethnic groups, it was found that the homozygous variant genotype *MTHFR* 677TT confers a reduced risk of CRC by 12%, but the risk between carriers of 677CT and CC genotypes was similar (Kennedy et al., 2012). On the other hand, a meta-analysis by Teng and colleagues in 2013 done on 71 studies and over 30 thousand patients showed an increased risk of CRC in carriers of the 677TT genotype, but only in Caucasian patients and not Asians (Teng et al., 2013). The same results were found in the Indian and the Hungarian population, with the caveat that this effect was found only on patients with rectal cancer in Hungary, but not colon cancer (Wang et al., 2006; Komlósi et al., 2010). A recent paper by Alanov and colleagues in Azerbaijan showed no effect of this polymorphism on the risk of CRC (Aslanov et al., 2023).

In our group, there was a slightly higher frequency of *MTHFR* 1298C allele (0.75) than in controls (0.65), and AA homozygosity at the 1298 polymorphic site was more common in patients than in healthy controls. As with the C677T polymorphism, data about the effect of A1298C polymorphism are not consistent. In a study by Jiang and colleagues, carriers of the 1298C allele had a lower risk of rectal cancer (Jiang et al., 2005). Same association was found in the Indian study where carriers of the 1298AC genotype were at a lower risk for colon cancer (OR 0.43, 95% CI 0.22–0.82) and rectal cancer (OR 0.7), and carriers of the homozygous CC genotype had a significantly lower risk for both colon (OR 0.3, 95% CI 0.09–0.80) and rectal cancer (OR 0.43, 95% CI 0.23–0.80) (Wang et al., 2006). This data would suggest a protective effect of the C allele of the A1298C polymorphisms. Again, a 33% reduction in the risk of rectal cancer in individuals carrying the 1298CC genotype was reported (Murtaugh et al., 2007). On the other hand, a Japanese case-control study on 220 patients with rectal cancer and controls found no association between this polymorphism and risk of rectal cancer nor did the meta-analysis of Kennedy and colleagues (Matsuo et al., 2005). However, in this meta-analysis, there was a lower risk of CRC in carriers of the 1298CC genotype in Asian and American studies, but an increased risk of CRC in European countries, which would suggest some geographic differences, possibly related to dietary habits of folate intake or alcohol consumption.

Very few studies stratified results also based on sex, but as before, results are inconclusive and inconsistent. Lightfoot et al. found a reduced risk of CRC in men with the 677CT genotype, and an increased risk in women with the 677TT genotype, while most other studies didn’t find any difference (Lightfoot et al., 2008). Komlosi et al. investigated the Hungarian population and stratified results according to age and sex, finding that the presence of the 677 C allele increased the risk for rectal cancer only in younger (<60) and male patients, but found no influence on colon cancer risk (Komlósi et al., 2010). On the other hand, Murtaugh et al. found a reduced risk of rectal cancer only in female carriers of the 677 T allele, especially in women 60 years or older (OR 0.32, 95% CI) in the United States (Murtaugh et al., 2007). Our analysis highlighted the protective role of the 677TT genotype for rectal cancer development in males (*p* = 0.030) and females (*p* = 0.044) as well as 1298CC in females (*p* = 0.017).

There is not much data on the combined effect of *MTHFR* C677T and A1298C polymorphisms and the risk of CRC. It has been previously described that the *MTHFR C677T* and *A1298C* polymorphisms are in linkage disequilibrium, which means that combinations of *677CT* and *1298CC*, *677TT* and *1298AC*, and *677TT* and *1298CC* are very rare (Kono and Chen, 2005). In this review, it was found that having one variant allele of either *677T* or *1298C* does not affect the risk of colorectal cancer. In the study by Murtaugh et al. the lowest risk of CRC was found in carriers of 677CC/1298CC. However, in the meta-analysis by Kennedy and the study of Yin et al. carriers of the 677TT/1298AA had the lowest risk of CRC, and carriers of 677CC/1298CC had a non-significantly higher risk of CRC in Yin`s study, again, showing inconsistencies in literature data (Yin et al., 2004). Our results suggest that individuals with haplotypes 677C and 1298A have an increased risk for rectal cancer development compared to any other haplotype while 677T and 1298C haplotypes have a protective role. Combined genotype analysis highlighted that individuals with the CC/AA genotype combination have an increased risk for rectal cancer development while the CT/AC genotype has a protective role.

Although our analysis did not show a statistically significant association between *MTHFR* C677T and A1298C polymorphisms and response to therapy, our results are somewhat different than most previously reported in the literature. We found a slightly lower frequency of the 677C allele in responders (0.68) than in non-responders (0.76), suggesting that carriers of the C allele are less likely to respond to nCRT. Most other studies (Longley et al., 2003; Vecchio et al., 2005; Balboa et al., 2010; Cecchin et al., 2011; Garcia-Aguilar et al., 2011; Thomas et al., 2011; Ulrich et al., 2014; Zhao et al., 2015; Salnikova and Kolobkov, 2016) found a relationship with the 677C allele and better response to nCRT, which goes further to suggest a very complex and probably multifactorial influence of genetic and other factors on the efficacy of nCRT. The most frequent explanation of the effect of *MTHFR* polymorphisms on the efficacy of 5FU-based nCRT is that the decreased activity of MTHFR causes elevated levels of 5,10-MTHF, thus reducing TS activity and disrupting DNA and RNA synthesis (Longley et al., 2003; De Mattia et al., 2020).

However, another hypothesis focuses on the higher availability of non-methylated folate substrates in patients with reduced MTHFR activity, which can be used for *de novo* synthesis of DNA, thus preserving DNA integrity. Also, carriers of the 677TT genotype could be less prone to DNA damage by radiotherapy, thus making the nCRT less effective in these patients (Kawakami et al., 2003; Leopardi, 2006).

All this emphasizes the complexity and multifactorial influences on treatment outcomes, especially in patients treated with combined modalities.

Although this study indicated the importance of *MTHFR* gene polymorphisms for the risk of developing locally advanced rectal cancer, it has some limitations. The main limitation of the study is the relatively low number of samples, although it is a representative sample of the Serbian LARC population (Flikkema and Toledo-Pereyra, 2012). The single-center and retrospective nature of the study are also limitations, thus validation of our findings and further survival analysis is planned to be performed on a prospective cohort whose collection is in progress at our Institute.

## 5 Conclusion

Data obtained in this study points to low-cost, non-invasive, and easily determined factors that might be helpful in identifying specific subgroups of patients that should be monitored more closely, which is especially important in developing countries. The *MTHFR* 667C allele and 1298A alleles were identified as low-penetrance risk factors for rectal cancer in our population. Our study did not show the influence of these polymorphisms on the efficacy of nCRT, but a further and more detailed analysis using a wider panel of genetic factors is planned. To the best of our knowledge, this is the first study of its kind done on the Slavic population in the Western Balkan region. Knowing that a variety of population-based characteristics may also be significant in this setting, this study could be helpful for future meta-analyses and used for construction of genetic cancer risk prediction panels.

## Data Availability

The original contributions presented in the study are included in the article/Supplementary material, further inquiries can be directed to the corresponding author.
